# Therapeutic Roles of Heme Oxygenase-1 in Metabolic Diseases: Curcumin and Resveratrol Analogues as Possible Inducers of Heme Oxygenase-1

**DOI:** 10.1155/2013/639541

**Published:** 2013-09-11

**Authors:** Yong Son, Ju Hwan Lee, Hun-Taeg Chung, Hyun-Ock Pae

**Affiliations:** ^1^Department of Anesthesiology and Pain Medicine, Wonkwang University School of Medicine, 460 Iksandae-ro, Iksan 570-749, Republic of Korea; ^2^Department of Biological Science, University of Ulsan, 30 Daehack-ro, Ulsan 680-749, Republic of Korea; ^3^Department of Microbiology and Immunology, Wonkwang University School of Medicine, 460 Iksandae-ro, Iksan 570-749, Republic of Korea

## Abstract

Metabolic diseases, such as insulin resistance, type II diabetes, and obesity, are associated with a low-grade chronic inflammation (inflammatory stress), oxidative stress, and endoplasmic reticulum (ER) stress. Because the integration of these stresses is critical to the pathogenesis of metabolic diseases, agents and cellular molecules that can modulate these stress responses are emerging as potential targets for intervention and treatment of metabolic diseases. It has been recognized that heme oxygenase-1 (HO-1) plays an important role in cellular protection. Because HO-1 can reduce inflammatory stress, oxidative stress, and ER stress, in part by exerting antioxidant, anti-inflammatory, and antiapoptotic effects, HO-1 has been suggested to play important roles in pathogenesis of metabolic diseases. In the present review, we will explore our current understanding of the protective mechanisms of HO-1 in metabolic diseases and present some emerging therapeutic options for HO-1 expression in treating metabolic diseases, together with the therapeutic potential of curcumin and resveratrol analogues that have their ability to induce HO-1 expression.

## 1. Introduction

The clustering in an individual of multiple metabolic abnormalities associated with metabolic diseases, including insulin resistance (IR), type II diabetes (T2D), and obesity, is defined as the metabolic syndrome (MS) [1]. It is well accepted that MS increases the risk of developing cardiovascular disease (CVD) [2]. Although there is a therapeutic treatment to combat some of metabolic diseases, especially T2D and CVD, both the intake of proper diets and maintaining healthy lifestyles are considered the best preventive measures [3]. However, for patients with MS, it is difficult to follow a diet/exercise regime that would improve their symptoms. Therefore, the identification of agents that may deal with more serious aspects of MS is an important medical field for research. Numerous experimental studies have confirmed the important role of naturally occurring phytochemicals in prevention and treatment of metabolic diseases [4]. Curcumin (Cur), resveratrol (Res), and their related derivatives are the most studied compounds in these fields so far [5]; therefore, we will discuss the therapeutic usage of Cur and Res in the context of metabolic diseases, together with the underlying mechanisms of action.

Recent studies have suggested that almost all of metabolic diseases are associated with a low-grade chronic inflammation (hereafter referred as to inflammatory stress), oxidative stress, and endoplasmic reticulum (ER) stress, and the integration of these stresses is critical to the pathogenesis of metabolic diseases [6]. Moreover, these stresses may interact with each other and amplify during pathogenesis of metabolic diseases. Thus, agents and cellular molecules that can reduce these stresses are emerging as potential targets for intervention and treatment of metabolic diseases.

Heme oxygenase-1 (HO-1), a ubiquitous inducible cellular stress protein, serves a major metabolic function as the rate-limiting step in the oxidative catabolism of heme, leading to formation of equimolar amounts of biliverdin (BV), free iron, and carbon monoxide (CO) [7]. BV formed in this reaction is rapidly converted to the strong antioxidant bilirubin (BR), which is then converted back into BV through the actions of reactive oxygen species (ROS) [8]. This cycle allows for the neutralization of ROS, which is considered as one of the antioxidant functions of HO-1 [8]. Besides heme degradation, HO-1 has been shown to exert other biological activities that play important roles in cellular protection [9]. The protective biological activities conferred by HO-1 include antioxidant, anti-inflammatory, and antiapoptotic effects [9]. By virtue of these protective activities, HO-1 has been suggested to play important roles in pathogenesis of metabolic diseases [10]. In this review, we will explore our current understanding of the protective mechanisms of HO-1 in metabolic diseases and present some emerging therapeutic options for HO-1 expression in treating metabolic diseases, together with the therapeutic potential of Cur and Res.

## 2. Metabolic Stress

A growing body of evidence suggests an early and central role of increased systemic oxidative stress as causal pathways linking with metabolic diseases [11]. In addition to oxidative stress, inflammatory stress and ER stress are also present in the patients with metabolic diseases [6]. Although a role for individual processes, such as oxidative stress, ER stress, and inflammatory stress, in metabolic diseases has been recognized in scattered reports, how these processes are interrelated in bringing about metabolic diseases has not been clear. However, these processes are ultimately integrated in the pathogenesis of metabolic diseases, which is referred as to metabolic stress. 

### 2.1. Oxidative Stress in Metabolic Diseases

The ROS of which production is an unavoidable consequence of aerobic metabolism in animal cells consist primarily of the various oxygen free radicals, including superoxide anion radical (O_2_^•−^) and hydroxyl radical (HO^•^), as well as the potent oxidizing molecules, including hydrogen peroxide (H_2_O_2_). At their high concentrations, ROS can react with many different macromolecules, thereby causing damage to, for example, DNA, proteins, and lipids [12]. ROS, therefore, play a major role in many disease processes. Despite their destructive activity, low/moderate levels of ROS are indispensable in several biochemical processes, including intracellular messaging and defense against microorganisms [13]. Thus, it is necessary for the cells to control the levels of ROS tightly to avoid any oxidative injury and not to eliminate them completely. This is supported by the fact that levels of ROS are tightly regulated by cellular antioxidant defense systems including small antioxidant molecules, such as glutathione (GSH), and ROS-scavenging enzymes, such as superoxide dismutase (SOD), catalase, and glutathione peroxidase (GPX) [14]. In this regard, oxidative stress has been shown to describe a condition in which these cellular antioxidant defense mechanisms are insufficient to inactivate ROS, or excessive ROS are produced, or both. Oxidative stress has been implicated in the development of IR and subsequent T2D [15]. 

One of the main cellular organelles involved in the production and regulation of ROS levels is the mitochondria, where superoxide is generated through electron transport chain (ETC) and converted to hydrogen peroxide either spontaneously or by SOD. In a state of chronic nutrient/energy overload, the flux of nutrients through the mitochondrial ETC can be increased, thereby enhancing ROS production and eventually inducing oxidative stress. ROS have been hypothesized to inhibit the cell signaling of the insulin receptor by blocking the pathway between insulin-receptor substrate 1 (IRS-1) and phosphatidylinositol-4,5-bisphosphate 3-kinase (PI3K), thereby inducing IR [16]. This hypothesis has been supported by the findings demonstrating that IR animal models are characterized by persistently elevated ROS levels [17]. In these animal models, pharmacological or genetic strategies designed to decrease ROS levels at least partially prevent IR status [17]. However, ROS are also necessary to maintain normal insulin sensitivity, which was underscored by the findings that mice lacking the antioxidant GPX showed higher ROS levels and enhanced insulin sensitivity [17]. Interestingly, treatment of mice lacking GPX with an antioxidant actually made the glucose metabolism worse. It is not clear why in certain cellular and animal models IR is associated with increased ROS levels, whereas in other models high ROS levels are associated with improved insulin sensitivity.

### 2.2. Inflammatory Stress in Metabolic Diseases

The metabolic diseases, including obesity and IR, are associated with a low-grade inflammation characterized by overexpression of proinflammatory cytokines produced by the expanding adipose tissue, activated macrophages, and other immune cells [18]. Inflammatory mediators, such as tumor necrosis factor-*α* (TNF-*α*), interleukin-1*β* (IL-1*β*), IL-6, leptin, resistin, monocyte chemotactic protein 1, plasminogen activator inhibitor-1, C-reactive protein (CRP), fibrinogen, angiotensin, visfatin, retinol binding protein-4, and adiponectin, can affect the metabolic functions of several organs, including the liver, heart, muscle, and brain [18], and some of them, especially TNF-*α* and IL-1*β*, can impair insulin signaling in insulin-responsive organs, which, as such a result, causes systemic IR [18]. In fact, the elevated levels of proinflammatory cytokines are detected in patients with the IR-associated clinical states and in experimental mouse models of obesity [19–21]. The exact mechanism by which proinflammatory cytokines can induce local and systemic IR is as yet unclear. However, several potential mechanisms for metabolic effects of TNF-*α* and IL-1*β* have been described. TNF-*α* stimulates serine kinases, such as c-jun *N*-terminal kinase (JNK) and p38 mitogen-activated protein kinase (MAPK) [22]. Activation of JNK and/or p38 MAPK by TNF-*α* results in serine phosphorylation of IRS-1 and IRS-2, which in turn reduces the downstream insulin signaling [23]. In adipocytes, TNF-*α* induces IR by reducing the expression of glucose transporter 4 (GLUT4) and peroxisome proliferator-activated receptor-*γ* [24]. Similarly, prolonged IL-1*β* treatment reduces the insulin-induced glucose uptake in 3T3 adipocytes, which is associated with a slightly decreased expression of GLUT4 and marked inhibition of GLUT4 translocation to the plasma membrane in response to insulin [25]. 

Although the mechanisms causing inflammation during obesity are under investigation, it is now recognized that the immune sensors, including Toll-like receptors (TLRs), Nod-like receptors (NLRs), inflammasome, and other pathogen-sensing kinases, participate in the development of obesity-associated inflammation. Of these receptors, TLR4 has been shown to be activated by saturated fatty acids (FAs) to generate inflammatory signals in macrophages, endothelial cells, and adipocytes, which ultimately results in the production of proinflammatory cytokines, such as TNF-*α* and IL-1*β* [26]. The bacterial endotoxin lipopolysaccharide (LPS) is a classical ligand for TLR4 in most cell types. The majority of the biological activity of LPS is contained within a moiety that is acylated with saturated fatty acids, and removal of these fatty acids results in complete loss of its ability to activate TLR4, suggesting that there is a degree of similarity in structure among LPS and saturated FAs. The NLR family can also sense obesity-induced signals in multiple contexts. In macrophages, NLR activation stimulates the cryptopyrin/NLRP3 inflammasome to induce IL-1*β* and IL-18 production via caspase-1 activation [27]. Similar to their activation of TLR4, saturated FAs, such as palmitic acid (PA), have been linked to inflammasome activation in macrophages [28]. PA is a major component of dietary saturated fat, representing up to 20% of the total serum FAs. PA has been shown to be present in a high percentage of atherosclerotic lesions [29]. It has been demonstrated that in cultured endothelial cells (ECs), downregulation of IRS-1 signaling by PA is dependent on each of the key proteins in the TLR4 signaling pathway: TLR4, myeloid differentiation factor-88 (MyD88), interleukin-1 receptor-associated kinase (IRAK), inhibitory *κ*B-kinase *β* (IKK*β*), and nuclear factor-*κ*B (NF-*κ*B) [30, 31]. PA activates TLR4, which in turn engages MyD88 and IRAK, subsequently activating IKK*β* and NF-*κ*B. Activation of NF-*κ*B inhibits IRS-1 tyrosine phosphorylation via an as yet unidentified mechanism.

### 2.3. ER Stress in Metabolic Diseases

The ER, a membrane compartment located near the nucleus, is a highly dynamic organelle responsible for protein folding, maturation, quality control, and trafficking. This organelle also has an important role in Ca^2+^ storage and signaling. When the ER becomes stressed due to the accumulation of newly synthesized unfolded proteins, this condition has been referred to as an ER stress and the unfolded protein response (UPR) is activated to increase protein folding capacity and to decrease unfolded protein [32]. If these mechanisms of adaptation are insufficient to recover ER homeostasis, the UPR will induce cell death programs to eliminate the stressed cells and might subsequently contribute to disease states, such as diabetes and its complications. In animal cells, the UPR is mediated by at least three transmembrane proteins: inositol-requiring enzyme 1 (IRE1), protein-kinase-RNA-like ER kinase (PERK), and activating transcription factor 6 (ATF6) [33]. Under unstressed conditions, these transmembrane proteins are maintained in an inactive state by binding to the major ER chaperone, or immunoglobulin heavy chain binding protein/glucose-regulated protein 78 (BiP/GRP78), at the side of the ER lumen. During ER stress, BiP is displaced to interact with misfolded luminal proteins, resulting in the release of IRE1, PERK, and ATF6 and leading to their activation [34]. The activation of PERK can result in sequential phosphorylation of the *α* subunit of eukaryotic translation initiation factor 2 (eIF2*α*), leading to rapid reduction in the initiation of mRNA translation and ultimately reducing the load of new proteins in the ER. Phosphorylation of eIF2*α* by PERK also allows the translation of activating transcription factor 4 (ATF4) that can induce transcription of genes involved in amino acids synthesis and apoptosis, such as CCAAT/enhancer-binding protein homologous protein (CHOP) [32–34]. IRE1 activation unmasks its endoribonuclease activity that is responsible for the unconventional splicing of the X box-binding protein 1 (XBP1) mRNA and its translation into the transcription factor XBP1 protein. The XBP1 protein upregulates the transcription of genes encoding ER chaperones, phospholipid biosynthesis, and components of the ER-associated degradation (ERAD) machinery that can dispose misfolded proteins [32]. IRE1 also activates JNK by recruiting the scaffold protein tumor necrosis factor receptor-associated factor 2 as well as the apoptosis signal-regulating kinase and caspase-12 [33]. Once activated, ATF6 translocates from the ER to the Golgi, where it is cleaved by regulated intramembrane proteolysis by site 1 and site 2 proteases. The cytoplasmic part of ATF6, an active transcription factor, transactivates genes encoding ER chaperones, ERAD components, and protein foldases [6, 34]. 

A number of biochemical, physiologic to pathologic stimulus, such as those that cause ER calcium depletion, altered glycosylation, nutrient deprivation, oxidative stress, DNA damage, or energy perturbation/fluctuations, can interrupt the protein folding process and result in ER stress. Interestingly, high metabolic process and obesity, which are induced due to high nutritional intake, also result in ER stress which suppresses insulin signaling [6]. A study has demonstrated protection against obesity-induced T2D in mice by overexpression of ER chaperones, while knockdown of chaperones was diabetogenic [35]. Furthermore, animal treatment with chemical chaperones that alleviated obesity-induced ER stress led to improvement in insulin sensitivity [35]. The mechanism by which ER stress can induce IR is not clear. One possibility is that UPR activation may stimulate stress kinases (e.g., JNK) that interfere with insulin signaling, thereby promoting IR. Also, transcription factors activated by UPR may modify transcription of key enzymes involved in gluconeogenesis or lipogenesis, thereby participating to the abnormal activation of these pathways in IR states. Finally, it is also possible that ER stress may lead to an increase in oxidative stress and/or inflammatory stress that in turn may contribute to IR. 

## 3. The Role of HO-1 in Metabolic Diseases

**Figure 1:**
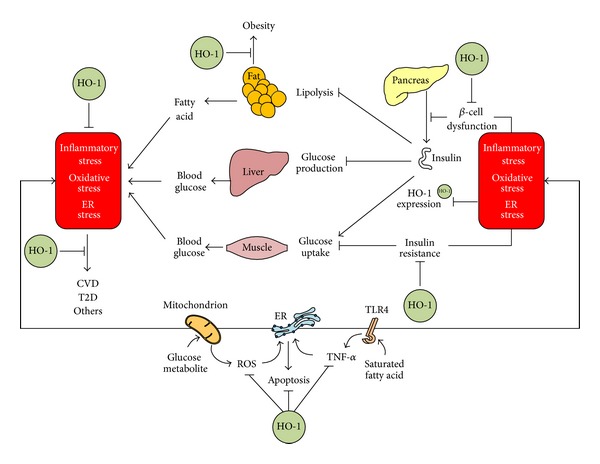
Therapeutic targets of HO-1 during pathogenesis of metabolic diseases. Metabolic diseases, such as CVD, T2D, and obesity, frequently arise from defects among coordinated actions of multiple tissues. Cells in a tissue may be exposed to oxidative stress generated mainly by mitochondria, inflammatory stress initiated probably by saturated FA-TLR4 interaction, and ER stress triggered by inflammatory and oxidative stresses, and these stresses, when prolonged, may amplify and integrate with each other. The integration of advanced stresses may cause one or more of metabolic diseases. HO-1 expression may reduce oxidative stress, inflammatory stress, and ER stress, thereby exerting therapeutic actions.

Although HO-1 is known initially for its role in heme catabolism, HO-1 has become increasingly recognized to exert a major role in cellular defense mechanisms [9]. The protective biological activities conferred by HO-1 include its antioxidant, anti-inflammatory, and antiapoptotic properties [9]. These protective effects of HO-1 are dependent on the generation of its enzymatic reaction products (i.e., CO, BV/BR). There is ample evidence that HO-1, in particular, can protect against metabolic diseases (Figure 1) [36–42]. 

### 3.1. HO-1 Expression

Targeted modulation of HO-1 expression for potential therapeutic interventions requires detailed knowledge of the mechanisms that can regulate HO-1 gene expression. The nuclear factor-erythroid 2-related factor 2 (Nrf2) is recognized as a major contributor to the upregulation of multiple antioxidant defense system in response to exogenous and endogenous stimuli or naturally occurring phytochemicals. Nrf2 belongs to the cap'n'collar family of basic region-leucine zipper-type transcription factors [7, 9, 32]. Nrf2 binds to the antioxidant-responsive element (ARE) or the electrophile-responsive element [7]. ARE has been detected in the promoter or upstream promoter regions of the genes encoding phase II antioxidant enzymes, including glutathione *S*-transferase subunits, glutamate-cysteine ligase catalytic and glutamate-cysteine ligase modifier subunits, the thioredoxin and peroxiredoxin families, and NAD(P)H:quinone oxidoreductase [7, 9, 32]. HO-1 is upregulated via activation of the Nrf2-ARE pathway. Several phytochemicals (Res, Cur, flavonoids, carnosol, etc.) and endogenous mediators can upregulate HO-1 expression via Nrf2-ARE pathway [9]. Nrf2 activation is mainly controlled by the cytosolic inhibitor Kelch-like enoyl-CoA-hydratase-associated protein1 (Keap1) [7, 9]. Under normal conditions, Nrf2 is anchored in the cytoplasm through binding to Keap1, which in turn facilitates the ubiquitination and subsequent proteolysis of Nrf2. Such sequestration and degradation of Nrf2 in the cytoplasm are mechanisms for the repressive effects of Keap1 on Nrf2. Keap1 contains two critical cysteine residues which are a second group of cysteines important for stress sensing. Disruption of the Nrf2-Keap1 complex can result from modification of critical cysteines of Keap1. Numerous stimuli cause disruption of the Nrf2-Keap1 complex via modulation of its critical cysteines, which permits subsequent nuclear translocation of free Nrf2. Thus, the Keap1/Nrf2 system appears to be a central sensor for a broad spectrum of unfavorable cellular conditions. 

### 3.2. HO-1 against Oxidative Stress

The antioxidant effect of HO-1 has been highlighted in HO-1-knockout mice. As compared with wild-type mice, the liver from HO-1-knockout mice shows higher levels of oxidized proteins and lipid peroxidation [43]. Moreover, peritoneal macrophages from HO-1-knockout mice, as compared with wild-type controls, exhibit increased ROS [44]. Similarly, cells from the human case of HO-1 deficiency showed increased sensitivity to oxidative injury [45]. Upregulation of HO-1 expression protects against oxidative stress-induced cell death [46, 47]. Thus, HO-1 expression plays a role to counteract oxidative stress. The specific mechanisms by which HO-1 can mediate antioxidant effect are not clear, but BV and BR, a byproduct generated during the heme catabolism, have been suggested as potential antioxidants. In fact, addition of BR to the culture medium was reported to markedly reduce the cytotoxicity produced by oxidants [8, 9]. Similarly, HO-1expression by the HO-1 inducer hemin increased the resistance against oxidative cell injury; notably, this protective effect occurred only in cells that were actively producing BR [48]. It is important to note that upregulation of HO-1 is often associated with increased ferritin [49], which sequesters redox-active iron, a toxic byproduct of heme degradation [9]. Considering that HO-1, as noted above, has an ability to reduce oxidative stress, it is not surprising that HO-1 appears to protect from the development of metabolic diseases, such as the diabetes that is consistently associated with increased oxidative stress [10]. 

### 3.3. HO-1 against Inflammatory Stress

The anti-inflammatory effect of HO-1 has been also highlighted in HO-1-knockout mice. As compared with wild-type mice, HO-1-knockout mice exhibited hallmarks of a progressive chronic inflammatory state [43]. Peritoneal macrophages from HO-1-knockout mice, as compared with wild-type mice, exhibited increased proinflammatory cytokines [44]. Similarly, a case of human HO-1 deficiency also exhibited hallmarks of a proinflammatory state [45]. Various naturally occurring phytochemicals, which are a group of antioxidant compounds and are currently investigated for their anti-inflammatory and anticancer activities, have been shown to provide antiinflammatory protection via HO-1 expression [50]. The specific mechanisms by which HO-1 can mediate anti-inflammatory effects are not clear, but CO has been suggested as a potential mediator. Studies have shown that administration of CO inhibited the production of LPS-induced proinflammatory cytokines, such as TNF-*α* and IL-1*β* [51], and increased LPS-induced expression of the anti-inflammatory cytokine IL-10 [52]. Several possible mechanisms have been postulated to explain the anti-inflammatory action of CO. CO modulated MAPK pathways, including p38 MAPK, ERK, and JNK pathways [9]. CO causes a general downregulation of proinflammatory cytokine production through p38 MAPK-dependent pathways and NF-*κ*B inactivation [51]. Given that obesity, IR, T2D, and many related cardiometabolic complications share a metabolic milieu characterized by elevated inflammatory and oxidative insults [53], HO-1 expression would suppress these insults by exerting anti-inflammatory and antioxidant effects.

### 3.4. HO-1 against ER Stress

Molecules involved in ER stress response have two opposing functions: adaptive or proapoptotic. ER stress-responsive molecules have an adaptive function in cells that are exposed to mild and transient stresses, whereas these molecules have a proapoptotic function in cells exposed to severe and chronic stress. Chronic ER stress triggered by chronic nutrient overload may decrease insulin signaling in cells with its receptor, resulting in IR, and may also induce apoptosis of pancreatic *β*-cells that can produce insulin. Thus, ER stress-responsive molecules may play an important role in insulin biosynthesis and IR. A study has shown that HO-1 expression was induced in response to ER stress-inducing chemicals, such as thapsigargin, homocysteine, and tunicamycin (TM), in smooth muscle cells (SMCs) [54]. Interestingly, exogenous application of CO inhibited apoptosis induced by ER stress-inducing agents in SMCs, which was associated with the downregulated expression of the proapoptotic proteins. In human ECs, HO-1/CO also inhibited ER stress-induced apoptosis via p38 MAPK-dependent inhibition of the proapoptotic CHOP expression [33]. These studies suggest that HO-1/CO can confer cytoprotection against apoptotic signals originating from ER stress-responsive molecules. In addition to its antiapoptotic effect, CO, as abovementioned, has been shown to downregulate the inflammatory response triggered by ER stress. TM, an ER stress inducer, could induce ER stress in the liver of mice, which was determined by the level of spliced XBP-1 mRNA, an indicator of the ER stress response, and increased the levels of CRP, a marker of inflammation; high levels of CRP are linked to the development of cardiovascular disease and T2D [55]. A water-soluble CO donor reduced the levels of spliced XBP-1 mRNA, alone with a remarkable downregulation of CRP mRNA expression in TM-treated mice. The injection of a CO donor also suppressed ER stress-induced CRP expression in the serum of TM-treated mice, which was similar to the results of the *in vitro* experiments where CO reduced TM-induced CRP expression in human liver cells. This study suggests that inflammation triggered by ER stress can be suppressed by HO-1/CO, providing evidence that HO-1/CO may potentially be used in therapeutic strategies designed to control inflammatory diseases related to ER stress. Collectively, because ER stress has been associated with a number of metabolic diseases [53], HO-1 expression that, as noted above, can reduce ER stress may have therapeutic potential as novel treatments of metabolic disorders. 

### 3.5. HO-1 against Metabolic Stress

A growing body of evidence now exists to support the view that there may be the integration of oxidative stress, inflammatory stress, and ER stress in metabolic diseases [53]. Depending on the cell type and physiological process, either oxidative stress, or inflammatory stress, or ER stress may be more prominent or upstream of the others. However, these signaling pathways may interact and be ultimately integrated in the pathogenesis of metabolic diseases. Given the integration of oxidative stress, inflammatory stress, and ER stress, targeting only one of them may not be effective in controlling disease pathogenesis. As abovementioned, HO-1 has its potential ability to modulate oxidative stress, inflammatory stress, and ER stress, and this may explain why HO-1 expression could be effective in controlling metabolic diseases (Figure 1). The important changes that are observed after increased expression of HO-1 in obese and diabetic animal models include (1) prevention of weight gain, (2) reduction of inflammatory cytokines levels, (3) restoration of normal insulin sensitivity, and (4) improved vascular reactivity.

A sustained increase in HO-1 expression may ameliorate IR and compensatory hyperinsulinemia. It has been demonstrated that systemic induction of HO-1 by treatment with the HO-1 inducer, hemin, or cobalt protoporphyrin (CoPP) in ob/ob mice or Zucker diabetic rats reduced adiposity and improved insulin sensitivity [56, 57]. The protective effect of systemic HO-1 induction was attributed to an increase in adiponectin expression, enhanced AMP kinase (AMPK) activation in both adipocytes and skeletal muscles, and suppression of adipogenesis and inflammatory cytokine expression. It has been also demonstrated that adipocyte-specific overexpression of HO-1 attenuated high fat- (HF-) mediated adiposity and vascular dysfunction, increased insulin sensitivity; and improved adipocyte function by increasing adiponectin and by decreasing inflammatory cytokines [58]. These effects are reversed by the HO activity inhibitor, stannous mesoporphyrin, suggesting that HO-1 plays important roles in mediating such effects.

HO-1 expression may prevent the development of obesity in metabolic diseases. Administration of CoPP resulted in sustained body weight loss between 20 and 25% compared with rats receiving vehicle [59]. Actions of systemic CoPP administration on body weight are dependent on HO-1 expression, as evidenced by studies that have demonstrated that coadministration of an HO inhibitor significantly attenuates weight loss in male ob/ob mice [56]. Treatment with CoPP has been demonstrated to lower body weight in leptin receptor-deficient Zucker diabetic fatty rats [60]. While it is clear that induction of HO-1 both centrally and systemically can prevent the development of obesity, the mechanism by which HO-1 expression elicits weight loss is not known.

## 4. HO-1 Inducers and Their Therapeutic Potential

HO-1 may be protective against stress-associated physiological disorders on the basis of its rapid upregulation under various stress conditions and potent physiological regulating properties. Therefore, HO-1 expression has been suggested to have a general adaptive response and enhanced resistance to various stresses [9]. Some studies have suggested that HO-1 expression is downregulated in abnormal metabolic states and that HO-1 overexpression may ameliorate metabolic diseases [61, 62]. For example, compared with Zucker lean rats, Zucker obese rats showed a decrease in HO-1 expression and an increase in the proinflammatory TNF-*α* and IL-6 levels, and treatment of Zucker obese rats with the HO-1 inducer CoPP increased HO-1 expression, which was associated with a decrease in superoxide levels and TNF-*α* and IL-6 levels and an increase in plasma adiponectin, as compared with untreated controls [61]. This treatment also decreased the visceral and subcutaneous fat content and reduced weight gain. A study has explored the vascular cytoprotective effects of HO-1 against hyperglycemia-induced oxidative stress in experimental diabetes and found that vascular extracellular SOD and plasma catalase activities were significantly reduced in diabetic rats compared with nondiabetic rats and that upregulation of HO-1 expression by administration of CoPP caused a large increase in extracellular SOD levels [62]. In addition, aortic ring segments from diabetic rats exhibited a significant reduction in the vascular relaxation response to acetylcholine, which was reversed by CoPP administration [63]. The results of an *in vivo* study have provided support for the protective effects of HO-1 on islet cells, as administration of CoPP upregulated HO-1 expression in the pancreas, preserved *β*-cell numbers in the islets, and decreased blood glucose levels to normal in nonobese diabetic mice compared with untreated controls [64]. Accordingly, pharmacological expression of HO-1 may be a novel therapeutic intervention for metabolic diseases. Many phytochemicals, which have reported antioxidant and anti-inflammatory properties, could be explored for their potential to reverse oxidative stress, inflammatory stress, and ER stress, which may be finally useful for management of metabolic diseases. 

### 4.1. Cur Analogues as HO-1 Inducers

**Figure 2:**
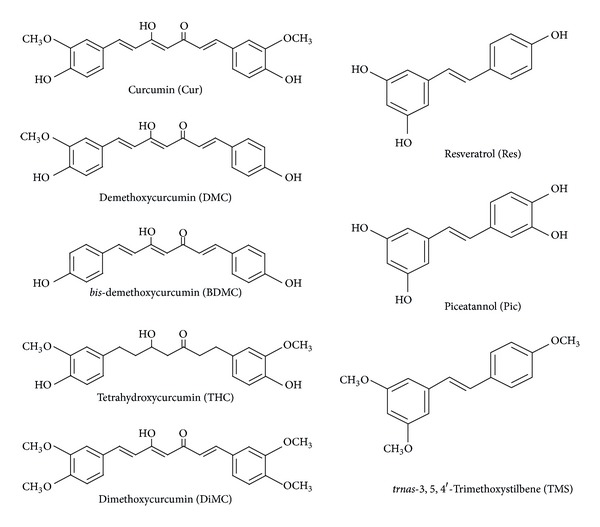
Chemical structures of Cur and Res analogues.

Turmeric is prepared by grinding dried rhizomes of *Curcuma longa*. Traditionally, turmeric has been used as a foodstuff and has been an important component of Indian medicine and traditional Chinese medicine [65]. Curcuminoids are the active components responsible for the majority of the medicinal properties of turmeric, and there are 3 naturally occurring curcuminoids: Cur, demethoxycurcumin (DMC), and *bis*-demethoxycurcumin (BDMC). Tetrahydrocurcumin (THC) is one of the major metabolites of Cur, and dimethoxycurcumin (DiMC) is one of synthesized Cur derivatives with metabolic stability over Cur. The chemical structures of Cur analogues are shown in Figure 2. While Cur contains two methoxyl groups at its *ortho*-position, DMC contains only one and BDMC contains none. In comparison with Cur, DiMC contains additional two methoxyl groups instead of two hydroxyl groups, and THC, like Cur, contains two methoxyl groups and two hydroxyl groups but lacks conjugated double bonds in the central seven-carbon chain. Cur has been first reported to induce *in vitro* HO-1 expression through Nrf2/ARE pathway in renal epithelial cells [66], which was further confirmed in rat vascular SMCs [67]. The *α*, *β*-unsaturated carbonyl group appears to be an important structure of curcuminoids, because THC, lacking this functional group, was virtually inactive in inducing HO-1 expression [68]. In fact, compounds carrying this reactive group have been reported to induce HO-1 expression through Nrf2 nuclear translocation [66]. It has been noted that three naturally occurring curcuminoids vary in their ability to induce HO-1 expression in human ECs [69]. The level of HO-1 expression was found to be highest with Cur, followed by DMC and BDMC. Considering that the main difference among the three curcuminoids is the number of methoxyl groups (none for BDMC, one for DMC, and two for curcumin), the presence of methoxyl groups in the *ortho*-position on the aromatic ring has been suggested to be essential to enhance HO-1 expression [69], and this finding may be useful in designing more efficacious HO-1 inducers. Cur is rapidly metabolized *in vivo* into THC and other reduced forms [70]. Moreover, HO-1-inducing property of Cur is lost when it is reduced to THC [67, 68]. Thus, there would be a need to develop Cur analogues with higher metabolic stability than the original Cur. DiMC, one of several synthetic Cur analogues, was reported to have increased metabolic stability in comparison with Cur [71], and, similar to Cur, induced HO-1 expression via Nrf2 activation in RAW264.7 macrophages [68]. Recently, a novel water soluble Cur derivative (NCD) has been developed to overcome low *in vivo* bioavailability of Cur and to evaluate its therapeutic effects in rats with diabetes mellitus induced by STZ [72]. Administration of oral NCD or pure Cur to diabetic rats significantly decreased blood glucose levels and increased the plasma insulin, as compared with the diabetic group, and NCD was more effective in such effects than Cur. Oral NCD did not change the plasma glucose levels in the control group, while it significantly increased the plasma insulin in the control group. Interestingly, treatment of diabetic rats receiving oral NCD with the HO-1 inhibitor zinc protoporphyrin resulted in a significant increase in the plasma glucose level and a significant decrease in insulin levels, when compared with the diabetic group receiving oral NCD only, and this strongly suggests that the antidiabetic effects of NCD might result from its activation of HO-1 but not from its activation of other antioxidant enzymes, such as SOD and catalase. Administration of oral NCD or pure Cur significantly increased the HO-1 expression level in the pancreatic tissues of the diabetic group, as compared with controls. Thus, it was suggested that the hypoglycemic action of Cur might be mediated through HO-1 expression. 

### 4.2. Res Analogues as HO-1 Inducers

Res, which is a plant polyphenol abundant in the skin of red grapes and also found in berries and peanuts, has been reported to induce HO-1 expression via Nrf2/ARE activation in neuronal PC12 cells [73]. It has been also reported that Res acts on the adipocyte to decrease the incorporation of lipids into adipocytes and retard the conversion of glucose to lipids [74]. Obese Zucker rats that were fed Res had lower blood pressure, plasma glucose, triacylglycerols, cholesterol, free fatty acids, leptin, and liver weight than animals that did not consume Res [75]. In another study, normal rats given a high-fat diet and Res had lower glucose and better insulin levels than rats fed a high-fat diet without the supplement [76]. In a diabetic rat model, rats that had a diet supplemented with Res had lower serum glucose, increased plasma insulin, normalized levels of carbohydrate metabolism enzymes, and lower hepatic proinflammatory cytokine levels [77]. Although many *in vivo* studies have demonstrated that HO-1 expression in specific tissues was induced by administration of Res, it is not certain whether Res could exert its beneficial effects directly by inducing HO-1 expression in targeted tissues. A study has examined whether HO-1 expression by Res could increase serum adiponectin levels and ameliorate vascular dysfunction in diabetic animals [78]. Administration of Res or CoPP increased serum levels of adiponectin in STZ-induced diabetic rats. Both Res and CoPP increased HO-1 expression in the aorta, compared to untreated diabetic rats. Interestingly, CO, one of HO-1 byproducts, which was released by a CO donor, also increased adiponectin levels, indicating a direct involvement of HO-1 activation. The increase in adiponectin was associated with a significant decrease in EC death. Res treatment in hypercholesterolemic rats also enhanced *in vivo* HO-1 expression, which plays an important role in neovascularization of the hypercholesterolemic myocardium [79]. In another study, treatment with Res decreased the blood glucose level and increased HO-1 expression, when compared to STZ-induced diabetic rats [80]. It is worthwhile to point out that the pharmacokinetic properties of Res are not favorable since Res has poor bioavailability being rapidly and extensively metabolized and excreted [81], which casts doubt on the physiological relevance of the high concentrations typically used for *in vitro* experiments. Nevertheless, a number of studies have demonstrated that Res was capable of exerting some of its beneficial effects *in vivo*, as abovementioned. Thus, it is most likely that one of Res metabolites may mimic some of *in vitro* beneficial effects of Res. Piceatannol (Pic) is a naturally occurring analog of Res and Pic has been also identified as one of Res metabolites [81]. Because Pic is generated during phase I metabolism of Res by cytochrome P450 enzymes and represents one of its main phase I metabolites [81], it was, therefore, hypothesized that Pic may have biological activities similar to those of Res. Pic possesses an additional hydroxyl group at Res structure (Figure 2). Pic, partly due to such a difference in the chemical structure, has a stronger effect on HO-1 expression than Res [82]. Interestingly, *trans*-stilbene with no hydroxyl groups and the semisynthetic trimethoxy-*trans*-stilbene (TMS) with methoxyl groups in lieu of the hydroxyl groups (Figure 2) have been found to be inactive in HO-1 expression [83]. In this regard, the hydroxyl groups of Res appear to be important for HO-1 expression. TMS, by reinforcing the hydrophobic character of the molecule and so potentially its diffusion through cellular membranes, loses its capacity to induce HO-1 expression but presents other effects [84]. More mechanistic studies are needed to evaluate and potentially confirm the beneficial effects of Res and its derivatives as an additional therapeutic approach by treating metabolic diseases.

### 4.3. Other HO-1 Inducers

Metalloporphyrins, such as CoPP and hemin, which are prototypical inducers of HO-1 and are commonly used in experimental cell culture and animal models, do not seem to be applicable for clinical interventions, because they lack cell-specificity and are severely toxic when it is used for long time periods. For example, CoPP suppresses thyroid and testicular hormone concentrations in serum, affects copper metabolism, elevates plasma ceruloplasmin levels, reduces hepatic cytochrome P450 levels, and has many other side effects [85–87]. By contrast, upregulation of HO-1 expression by natural dietary components widely present in food and nutraceuticals, such as Cur and Res, may exert beneficial effects under normal and pathological experimental conditions. A major advantage to the medical use of these compounds is that the dosage of each compound in a particular formulation needed to achieve a particular health-related effect is likely to be far below the range in which the compound may be toxic. Therefore, these substances would be an alternative for a possible HO-1 expression in humans. In addition, a number of currently available pharmacologic compounds, which can induce HO-1 expression and are applied in standard therapies, may be also useful for clinical interventions in metabolic disorders. 

Although potentially many phytochemicals may qualify to serve as HO-1 inducers, Cur and Res appear to have been tested most commonly in the literature. Besides Cur and Res, quercetin, a polyphenol found in a variety of fruits and vegetables, has been also reported to induce *in vitro* HO-1 expression via MAPK/Nnf2 pathway [88]. Overweight Zucker rats and normal-weight rats given quercetin had significantly lower glucose, triacylglycerols, and free fatty acids and higher insulin levels than rats that received no quercetin [89], suggesting its potential to treat metabolic diseases. However, whether quercetin, by inducing *in vivo* HO-1 expression, could ameliorate the risk factors that lead to the development of metabolic diseases remains to be investigated. 

## 5. Conclusions

There is increasing evidence that complications related to metabolic diseases are associated with elevated oxidative stress, inflammatory stress, and ER stress (Figure 1) [6]. The integration of these stresses plays an important role in the pathogenesis and development of metabolic diseases [6]. HO-1 expression has been shown to be protective against metabolic diseases, at least in part, by reducing these stress responses, and this has generated immense interest in HO-1 as a therapeutic target. Naturally occurring phytochemicals ameliorate the risk factors that lead to the development of metabolic diseases, but the mechanisms of their actions remain to be established. In animal models, some of them, such as Cur and Res, reduce the incidence of metabolic diseases via Nrf2-dependent HO-1 expression [72, 78–80], which allows them to be considered as HO-1 inducers that may provide an alternative strategy for controlling the initiation and progression of metabolic diseases. However, their introduction into the clinical setting may be hindered largely by their poor solubility, rapid metabolism, or a combination of both, ultimately resulting in low therapeutic concentrations at the target site. To overcome the bioavailability, advanced drug delivery systems, designed to provide localized or targeted delivery of these agents, may provide a more viable therapeutic option in the treatment of metabolic diseases.
